# Role of Long Non-Coding RNA Polymorphisms in Cancer Chemotherapeutic Response

**DOI:** 10.3390/jpm11060513

**Published:** 2021-06-04

**Authors:** Zheng Zhang, Meng Gu, Zhongze Gu, Yan-Ru Lou

**Affiliations:** 1 School of Biological Science and Medical Engineering, Southeast University, Nanjing 210096, China; zheng@seu.edu.cn; 2 Department of Clinical Pharmacy and Pharmacy Administration, School of Pharmacy, Fudan University, Shanghai 201203, China; 20211030060@fudan.edu.cn; 3 State Key Laboratory of Bioelectronics, School of Biological Science and Medical Engineering, Southeast University, Nanjing 210096, China

**Keywords:** gene polymorphisms, cancer chemotherapy, drug response, drug toxicity, personalized oncology

## Abstract

Genetic polymorphisms are defined as the presence of two or more different alleles in the same locus, with a frequency higher than 1% in the population. Since the discovery of long non-coding RNAs (lncRNAs), which refer to a non-coding RNA with a length of more than 200 nucleotides, their biological roles have been increasingly revealed in recent years. They regulate many cellular processes, from pluripotency to cancer. Interestingly, abnormal expression or dysfunction of lncRNAs is closely related to the occurrence of human diseases, including cancer and degenerative neurological diseases. Particularly, their polymorphisms have been found to be associated with altered drug response and/or drug toxicity in cancer treatment. However, molecular mechanisms are not yet fully elucidated, which are expected to be discovered by detailed studies of RNA–protein, RNA–DNA, and RNA–lipid interactions. In conclusion, lncRNAs polymorphisms may become biomarkers for predicting the response to chemotherapy in cancer patients. Here we review and discuss how gene polymorphisms of lncRNAs affect cancer chemotherapeutic response. This knowledge may pave the way to personalized oncology treatments.

## 1. Background

Transcriptomic studies have implicated that up to 90% of eukaryotic genomes are transcribed [1], and the Human Genome Project revealed that only about 1.2% of the human genome encoded proteins, suggesting that a large number of transcripts were non-coding [1]. Non-coding RNAs with more than 200 nucleotides are defined as long non-coding RNAs (lncRNAs). The advent of advanced technologies, such as RNA-Seq and ChIP-Seq, has helped to identify the functions of lncRNAs in embryonic growth, pluripotency, cell cycle, cell differentiation, immune response, and disease development [2], while some of their functions are largely unknown. By interactions with DNA [3], RNA [4], and proteins [5], lncRNAs can participate in pathways of gene regulation. Recent studies have shown that lncRNAs seem to regulate their expression levels in a polymorphism-dependent manner and that they participate in different signal pathways, such as PI3K/AKT/NF-κB, thereby achieving the purpose of influencing the chemotherapy response. Some of them associated with imprinted regions have been extensively studied, such as *H19*, *IPW*, and *MEG3* [6]. Some well-studied lncRNAs have proved to play a role in epigenetic regulation. For example, *HOTAIR*, is located in the *human homeobox transcription factors C* (*HOXC*) locus, it interacts with polycomb repressive complex 2 (PRC2), thereby repressing the transcription at the *HOXD* site across 40 kilobases, which may have important implications for gene regulation in development and disease status [7]. Previous studies have shown that lncRNAs can interfere with the miRNA pathway as a sponge [8]. As a new regulatory mechanism, lncRNAs and miRNAs may also form a mutual inhibitory regulatory loop [9]. Additionally, lncRNAs also act as key signal transduction mediators in cancer signaling pathways via the regulation of differentiation, cell proliferation and apoptosis, invasion and metastasis, and chemoresistance [10,11], and alterations in these functions are associated with their gene polymorphisms. Genetic polymorphisms are defined as the presence of two or more different alleles in the same locus, with a frequency of higher than 1% in the population. Single nucleotide polymorphisms (SNPs) are defined as DNA sequence polymorphisms caused by a single nucleotide variation at the genomic level [12]. The genome-wide association study (GWAS) has recognized SNPs as the most common type of genetic variation in the human genome (one per 100–300 nucleotides) [13], associated with cancer tumorigenesis [14]. Recent studies have found that aberrant expression of specific lncRNAs was strongly associated with the earlier diagnosis or prognosis of patients with upper gastrointestinal tumors [14]. In fact, lncRNA-associated SNPs show a strong ability to interfere with the function of lncRNA-regulated genes, which are involved in important signaling pathways and carcinogenesis. Some polymorphisms in lncRNA *maternally expressed gene 3* (*MEG3*) are considered as well-established cancer biomarkers and therapeutic targets due to their association with cancer risk and response to chemotherapy [15]. In this review, we focus on the role of lncRNAs in cancers, particularly the role of lncRNA polymorphisms in response to anti-cancer therapies.

## 2. lncRNA Polymorphisms in Cancer Chemotherapeutic Response

In recent years, disease association studies have revealed the role of the SNPs of lncRNAs in various diseases. For example, the SNP of *SLEAR* rs13259960 was associated with susceptibility to systemic lupus erythematosus [16], and the SNP of *PART1* rs8176070 was associated with the risk of knee osteoarthritis [17]. Notably, lncRNAs SNPs can significantly affect gene expression and function, leading to alterations in cancer susceptibility, chemotoxicity, and sensitivity. For example, the *ANRIL* rs1333049 was thought to be associated with the risk of toxicity to platinum-based chemotherapeutic drugs in lung cancer patients [18]. *Long intergenic non-coding RNA for kinase activation* (*LINK-A*) rs12095274 might lead to resistance to serine/threonine kinase (AKT) inhibitors in breast cancer patients [19]. Compared with A allele carriers, rs2288947 G allele carriers have a significantly higher risk of bladder cancer susceptibility [20]. Chemotherapy, as one of the important methods of cancer treatment, is of great significance for prolonging the life of patients, but chemotherapy resistance that affects the effectiveness of chemotherapy is still inevitable. Fortunately, some recent studies on lncRNAs polymorphisms bring hope to the fight against chemotherapy resistance. These findings indicate that lncRNAs polymorphisms may have a potential meaning in personalized cancer therapy based on genotypes. Here, we summarize several studies of lncRNAs SNPs relevant to chemotherapy responses to further clarify the potential of lncRNAs as potential biomarkers of cancer risk and predictors of drug resistance as well as toxicity (Table 1).

### 2.1. MIR2052HG

**Figure 1:**
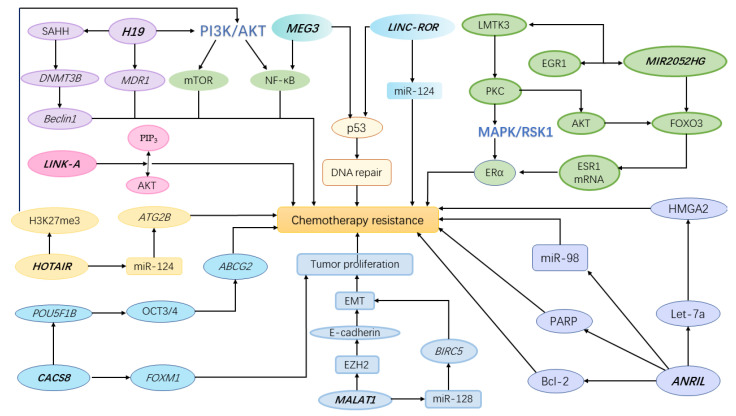
A schematic showing how polymorphisms affect the response to cancer chemotherapy. SAHH (S-adenosylhomocysteine hydrolase), *DNMT3B* (*DNA Methyltransferase 3 Beta*), *MDR1* (*multidrug resistance 1*), PIP_3_ (phosphatidylinositol 3,4,5-trisphosphate), *ATG2B* (*autophagy-related protein 2 homolog B*), *POU5F1B* (*POU Class 5 Homeobox 1B*), *FOXM1* (*Forkhead Box M1*), *ABCG2* (*ATP-binding cassette transporter G2*), *BIRC5* (*Baculoviral IAP Repeat Containing 5*), EMT (Epithelial-mesenchymal transition), *EZH2* (*Enhancer Of Zeste 2 Polycomb Repressive Complex 2 Subunit*), *HMGA2* (*High Mobility Group AT-Hook 2*), PARP (Poly(ADP-ribose) polymerase), Bcl-2 (B cell lymphoma-2).

*MIR2052HG*, located on chromosome 8q21.11, encodes a lncRNA. Previous research results suggested that *MIR2052HG* was related to estrogen receptor (ER)-positive breast cancer [28], which can be treated with aromatase inhibitors (AIs) by inhibiting the synthesis of estrogen [29]. Compared with 4406 healthy people, it was found that the level of MIR2052HG in 253 breast cancer patients increased, and this change in expression level was regulated by *MIR2052HG* SNPs [21]. GWAS analysis by Ingle et al. identified six SNPs that were associated with a longer breast cancer-free interval. Among them, rs3802201 was located in an intron of *MIR2052HG*, which could down-regulate the expression of *MIR2052HG* and made patients have a longer breast cancer-free interval [21]. In lymphoblastoid cell line (LCL) models, cells with this SNP showed estradiol dose-dependent up-regulation of *MIR2052HG* expression, which was reversed by an AI (exemestane or anastrozole) [21]. Strikingly, the expression of *ESR1* (encoding ERα) was correlated with the expression of *MIR2052HG* in these LCL models, which was in agreement with the analysis of The Cancer Genome Atlas data of 485 ER-positive breast cancer patients. It is common knowledge that the amplification of *ESR1* gene leads to the increased expression of ERα, which is associated with endocrine resistance [29]. This suggested that MIR2051HG may play a role in the resistance of breast cancer patients to AIs. Subsequently, by proteasome inhibitor and ubiquitin analysis experiments, Ingle et al. confirmed that *MIR2052HG* was involved in the degradation process of ERα in a ubiquitin-dependent and proteasome-mediated mode [21]. In addition, the researchers found that the down-regulation of *MIR2052HG* could reduce ESR1 mRNA expression by enhancing AKT-mediated decrease of forkhead box O3 (FOXO3), which was a regulator of ESR1 transcription. This finding indicates that the expression of *MIR2052HG* regulated the level of ERα at both the transcription level and the protein degradation level. Mechanistically, Cairns et al. confirmed that *MIR2052HG* promoted the transcription of lemur tyrosine kinase-3 (LMTK3) by interacting with early growth response protein 1 (EGR1) [22]. LMTK3 is related to new-onset and intrinsic endocrine resistance in breast cancer [30]. As a direct target of *MIR2052HG*, LMTK3 regulated downstream protein kinase C (PKC)/AKT/FOXO3 and PKC/MAPK/RSK1/ERα signaling pathways, thereby regulating the expression of ERα and the response to AI [21] (Figure 1). Notably, compared with the wild genotype, the stimulation effect of *MIR2052HG* SNPs on LMTK3 expression can be reversed by AIs. It showed that *MIR2052HG* regulated LMTK3 in a SNPs- and AI-dependent way [22]. In conclusion, the expression level of *MIR2052HG* was influenced by its genetic polymorphisms. Furthermore, different expression levels of *MIR2052HG* regulate the expression of ERα through transcription and protein degradation mode, which is related to the resistance of ERα-positive breast cancer patients to AIs.

### 2.2. MEG3

As a lncRNA, *Maternally Expressed Gene 3* (*MEG3*) located on the human chromosome 14q32.3 has an inhibitory effect in a variety of cancers, such as bladder cancer, gastric cancer, and non-small cell lung cancer [31,32,33]. In some pathways associated with cancer, such as TGF-β, PI3K/AKT, mTOR, and WNT/β-catenin pathways, *MEG3* plays a regulatory role. Previous studies have shown that *MEG3* was down-regulated in ER-positive breast cancer [34] and is also closely related to the chemotherapy response of breast cancer and colorectal cancer [23,35]. Ji et al. found that overexpression of *MEG3* increased the sensitivity of cisplatin-resistant cells to cisplatin, which was achieved by suppressing cell proliferation and inducing apoptosis [36]. In non-small cell lung cancer, up-regulation of *MEG3* led to an increase in the apoptosis rate of cancer cells. However, cancer cells with *MEG3* knockout have a decreased rate of cisplatin-induced apoptosis, and the result is that lung cancer cells enhance cisplatin resistance by activating the WNT/β-catenin signaling pathway [37]. In addition, researchers found that the *MEG3* polymorphisms may be a key factor for individual differences in chemotherapy toxicity and sensitivity to treatment [12]. By exploring the response of 505 patients with nasopharyngeal carcinoma after concurrent chemoradiotherapy treatment, it was found that patients with the *MEG3* rs10132552 CC genotype had a significantly higher risk of developing anemia (OR = 3.001, 95% CI = 1.355–6.646, *p* = 0.007), and patients with the rs10132552 CT genotype responded better to chemotherapy (OR = 0.261, 95% CI = 0.089–0.770, *p* = 0.015). In silico analysis results explained why *MEG3* polymorphisms could affect the sensitivity and toxicity of chemotherapy drugs in nasopharyngeal carcinoma patients. The local folding structure of RNA was altered when the T allele on rs10132552 was replaced by the C allele, which then affected the function of *MEG3*. Likewise, the results of the study by Bayarmaa et al. proved that *MEG3* rs10132552 was also related to chemotherapy response in breast cancer patients treated with paclitaxel or cisplatin [23]. In terms of mechanism, *MEG3* may inhibit the growth of cancer cells and induce their apoptosis by activating ER stress, nuclear factor-κB (NF-κB), and p53 pathways, and it may eventually affect the response to chemotherapy. Zhang et al. also believed that *MEG3* can regulate the expression of a variety of downstream genes of the p53 pathway [38], thereby regulating the proliferation and apoptosis of cancer cells. Additionally, *MEG3* genetic polymorphisms were also associated with platinum-based chemotherapy response in lung cancer [39]. In terms of chemotherapy toxicity, Gong et al. found that *MEG3* rs116907618 might play an important role in the occurrence of severe gastrointestinal toxicity caused by platinum drugs [18]. Particularly, linkage disequilibrium analysis showed that SNPs of rs10132552 and rs941576 had strong linkage [23]. Thus, the effect of SNP rs941576 on chemotherapy response can be further explored. Based on these results, *MEG3* polymorphisms could be used as a potential predictive biomarker for platinum-based adjuvant chemotherapy responses in relevant cancer patients. Moreover, it can also be used for individualized treatment of cancer patients to avoid serious adverse reactions of chemotherapy in some patients.

### 2.3. H19

*H19* plays a dual role of oncogene or tumor suppressor gene, depending on the cancer type and stage of development. In recent years, the impact of *H19* on cancer risk and prognosis has attracted researchers’ interest [18,40,41,42]. H19 has recently been found to be an independent posterior factor for low-grade gliomas [43]. As previously reported in the literature, *H19* polymorphisms were associated with the risk of several cancers. For instance, *H19* rs217727 was found to be associated with oral squamous cell carcinoma [44], osteosarcoma [45], bladder cancer [46], and gastric cancer [47] risk; *H19* rs2839698 has been shown to be associated with hepatocellular cancer risk and prognosis [48]. It was noteworthy that *H19* was overexpressed in many drug-resistant cancer cell lines, such as cisplatin-resistant ovarian cancer and doxorubicin-resistant breast cancer cells [25,49]. Recently, it was found that *H19* was overexpressed in methotrexate-resistant and 5-fluorouracil-resistant choriocarcinoma cells [50]. Methotrexate and 5-fluorouracil are the most important first-line chemotherapeutic agents. Further, studies substantiated that *H19* knockdown could reduce drug resistance in cells and promote drug-induced apoptosis in resistant choriocarcinoma cells. In terms of mechanism, the expression levels of *p*-PI3K, *p*-AKT, and *p*-mTOR proteins of *H19* knockdown were significantly lower than those of the *H19* non-knockdown group, and the PI3K/AKT/mTOR pathway may be the pathway by which *H19* exerts chemoresistance [50]. Notably, two *H19* SNPs (rs2107425 and rs2839698) were observed to be significantly correlated with platinum-based chemotherapy treatment in lung cancer patients [39]. Among them, *H19* rs2839698 T allele carriers had lower sensitivity to platinum-based chemotherapy drugs. In addition, the researchers found that *H19* rs2104725 and rs2839698 were both related to gastrointestinal toxicity in platinum-based chemotherapy reactions. Additionally, rs2839698 was also related to severe hematological toxicity [18]. Mechanically, *H19* regulates the methylation of the promoter of *multidrug resistance 1* (*MDR1*) gene, thereby inducing the expression of the product *p*-glycoprotein (encoded by *MDR1*) and *MDR1*-related drug resistance in human hepatocellular carcinoma HepG2 cells [42]. *MDR1* was also shown to be related to gastrointestinal toxicity caused by platinum-based chemotherapy [51]. Recent studies have found that the *H19*/SAHH/*DNMT3B* axis may be critical for tamoxifen resistance in breast cancer patients and then decreasing methylation in the promoter region of *Beclin1* [52]. These results corroborated that *H19* polymorphisms were related to the drug reaction and toxicity reaction of platinum chemotherapeutic drugs, but its molecular mechanism needs further study.

### 2.4. CASC8

The gene polymorphisms of *CASC8* have been reported to be closely related to the risk and progression of different cancers [53,54]. According to a recent study comparing TC and CC genotypes, gastric cancer patients carrying the rs1562430 TT genotype had a higher risk of death [55]. For rs10505477 TT genotype carriers, cancer risk was higher than TC or CC genotype carriers [56]. In a study of colorectal adenoma, two *CASC8* SNPs (rs10505477 and rs6983267) were found to be associated with colorectal adenoma risk, especially in patients without a family history of colorectal cancer [57]. Among them, rs10505477 A genotype carriers and rs6983267 G genotype carriers had a lower risk of developing adenoma. Based on these findings, more attention has been drawn to the effect of *CASC8* polymorphisms on chemotherapy responses. Shen et al. have proven that *CASC8* rs10505477 was related to the prognosis of gastric cancer patients receiving cisplatin chemotherapy after surgery [58]. Recently, through conducting chemotherapy response and toxicity studies on 467 patients, Gong et al. found for the first time that the SNP of *CASC8* rs10505477 was significantly correlated with platinum-based chemotherapy response (adjusted OR = 1.58, 95% CI = 1.05–2.39, *p* = 0.03) and toxicity (adjusted OR = 0.59, 95% CI = 0.35–0.98, *p* = 0.04), including severe hematological toxicity and gastrointestinal toxicity [27]. Prior to this, two studies have demonstrated that the *CASC8* gene location region overlapped with the *POU5F1B* gene, also known as *POU5F1P1* [58,59]. The protein encoded by *POU5F1P1* is functionally similar to the protein encoded by the *POU5F1* gene (also known as *OCT3* or *OCT4*) [60]. *OCT3/4* has been confirmed to be involved in the chemotherapy resistance of glioblastoma cell lines by affecting the expression of the drug efflux pump gene *ABCG2*, which encodes breast cancer resistance protein (BCRP) [61]. Non-small cell lung cancer patients with *ABCG2* overexpression are resistant to platinum-based chemotherapy [62]. Based on this evidence, Hu et al. surmised that the polymorphisms of *CASC8* rs10505477 could affect the efficacy and toxicity of platinum-based chemotherapeutics in patients with lung cancer by adjusting the *POU5F1B–OCT3/4-ABCG2* axis. Moreover, in non-small cell lung cancer cells, through Western blotting, researchers found that when *CASC8* was knockdown, the level of FOXM1 protein decreased [63]. *FOXM1* is an oncogene that promotes cell proliferation and promotes tumor development [64]. Subsequent studies substantiated that there was a positive regulatory relationship between *CASC8* and *FOXM1*. In the three cell lines (A549, H1299, and H460 cells), *CASC8* and FOXM1 were both increased in an osimertinib-dependent manner. Silencing *CASC8* can increase the sensitivity of NSCLC cells to osimertinib via *FOXM1*. These findings not only provide new insights for the clinical application of *CASC8* polymorphisms in predicting cancer risk and chemotherapy response but also provide new directions for exploring the effect of *CASC8* SNPs on chemotherapy.

### 2.5. LINK-A

*LINK-A*, also *LINC01139*, is a lncRNA with increased expression in cancer cell lines. A prior study suggests that highly expressed *LINK-A* may exhibit resistance to AKT inhibitors in breast cancer [19]. Since up-regulated expression of *LINK-A* in vivo is regulated by gene amplification and disease-related SNPs, researchers are beginning to focus on the effects of *LINK-A* SNPs on the development of drug resistance in cancer patients. By analyzing genetic mutations within or adjacent to the *LINK-A* gene locus in breast cancer patients, Lin et al. found that a SNP mutation located downstream of the *LINK-A* transcriptional region in breast cancer was associated with *LINK-A* expression and outcome [19]. In addition, meta-analysis corroborated that the expression of *LINK-A* was related to its genetic variation, and rs12095274 A allele carriers had higher *LINK-A* expression levels than G allele carriers. It is known that AKT and phosphatidylinositol 3,4,5-trisphosphate (PIP_3_) are involved in important signal transduction pathways in cells and play significant roles in cell growth. Therefore, the dysregulation of PI3K and the activation state of its downstream AKT are closely related to many cancers [65,66]. According to previous studies, lncRNA *LINK-A* can specifically interact with AKT and PIP_3_ in breast cancer cells [19]. *LINK-A* promotes the AKT–PIP_3_ interaction and the activation of downstream enzymes to cause tumor cell resistance to AKT inhibitors. Lin et al. found that the deletion of the PIP_3_-binding motif of *LINK-A* reversed the resistance of drug-resistant cells to MK2206 (an AKT inhibitor targeting the AKT pleckstrin homology (PH) domain). This is due to its inability to promote the binding of PIP_3_ to the PH domain of AKT [67]. Based on these studies, it can be believed that *LINK-A* rs12095274 A allele carriers are more likely than G allele carriers to develop resistance to AKT inhibitors in breast cancer patients. These genetic polymorphisms may result in poor treatment outcomes and may impact on survival in breast cancer patients. These studies suggest that *LINK-A* SNPs can be used as a marker to infer the sensitivity of breast cancer patients to AKT inhibitor therapy. Moreover, stratifying breast cancer according to the expression level of *LINK-A* is an effective way to achieve individualized treatment

### 2.6. Linc-ROR

*Linc-ROR*, is an effector of p53, but interestingly, it also has a regulatory effect on p53. The dysfunction of p53 function has been shown to relate to the occurrence and development of breast cancer [68]. Previous studies have suggested that *Linc-ROR* significantly inhibited p53 in the process of DNA damage, thereby affecting the arrest and apoptosis of cancer cells, which is believed to be the cause of resistance to platinum chemotherapy in cancer patients [24]. In addition, *Linc-ROR* has also been found to interact with miR-124 and to participate in the resistance of pancreatic cancer to gemcitabine by regulating the miR-124/PTBP1/PKM2 axis [69]. Wang et al. found that the *Linc-ROR* rs2027701 polymorphisms were associated with the poor efficacy of chemoradiotherapy in lymph nodes, but the mechanism was unclear [24]. Moreover, patients with rs2027701 in one or two variant alleles showed a clear trend of toxic reactions to platinum-based chemotherapy. A recent study confirmed that high *Linc-ROR* expression leads to doxorubicin resistance in hepatocellular carcinoma [70]. Combined with a finding by Luo et al. that the SNP of rs4801078 affects the expression level of *Linc-ROR* mRNA [71]. Therefore, it can be speculated that *Linc-ROR* SNPs may play an important role in chemoresistance by altering *Linc-ROR* mRNA’s expression level. These findings provide new insights into the function of *Linc-ROR* polymorphisms in cancer development and chemotherapy.

### 2.7. MALAT1

In recent years, lncRNA *MALAT1* has been proved to be a metastasis and prognosis marker of some cancers, and it was involved in the proliferation, invasion, and apoptosis of cancer cells [26,72]. The increased expression is positively correlated with cancer susceptibility and poor cancer prognosis. A prior study confirmed that mutation of one or two alleles from C to G on rs664589 increased the risk of colorectal cancer metastasis and carcinogenesis [73]. The change in the binding of *MALAT1* to miR-194-5p led to an increase in the expression level of *MALAT1* rs664589 in the G allele carrier, which leads to this outcome. In addition, the impact of *MALAT1* on chemotherapy response is also one of the focuses of researchers, especially its genetic polymorphisms. Lai et al. found that the silence of *MALAT1* significantly increased the sensitivity of cancer cells to cisplatin [74]. It has been suggested that rs619586 SNP may change *MALAT1* expression by affecting the transcription factor binding site [39], thus affecting chemotherapy toxicity. As one of the effective chemotherapeutic drugs for metastatic colorectal cancer, irinotecan has a wide range of inter-individual toxicity, which is related to the genetic characteristics of patients [75,76,77]. The resistance of tumor cells to irinotecan may be due to the inhibition of DNA topoisomerase I by *MALAT1* attenuated irinotecan. As evidence, in metastatic colorectal cancer, researchers found that increased levels of *MALAT1* of the cell cycle G1/S and M phases inhibited apoptosis and/or increased the efficiency of DNA repair [26]. Analogously, Li et al. proved that the up-regulation of *MALAT1* also led to a decrease in sensitivity to oxaliplatin [74]. Mechanically, *MALAT1* knockdown enhanced E-cadherin expression and inhibited oxaliplatin-induced EMT in colorectal cancer cells through *EZH2*. *EZH2* is a critical component of PRC2 [78], which silences E-cadherin during EMT and leads to cancer progression [79]. In addition, as a molecular sponge, *MALAT1* interacts with miR-218, also affecting chemotherapy response. After receiving standard FOLFOX treatment, patients with low *MALAT1* and high miR-218 expression had a higher survival rate than those with high *MALAT1* and low miR-218 expression [74]. In patients with high *MALAT1* expression, it was found that *MALAT1* binds *EZH2* to the CDH1 promoter and inhibits miR-218 during oxaliplatin treatment, indirectly promoting EMT, metastasis, and chemoresistance of colorectal cancer cells. Previous studies revealed that miR-218 could significantly suppress the EMT process and enhance 5-FU–based chemosensitivity in colorectal cancer cells by targeting *BIRC5*, a key member of the *inhibitors of apoptosis* gene (*IAP*) family [80,81]. *MALAT1* SNPs influenced the resistance of cancer cells to oxaliplatin through the regulation of their expression level. Moreover, researchers recently proposed that *MALAT1* polymorphisms were related to the drug resistance and toxicity of platinum-based chemotherapy drugs in lung cancer patients [82]. To sum up, the regulatory role of *MALAT1* polymorphisms in cancer chemotherapy is interesting, and its potential as a biomarker to predict the chemotherapy response of cancer patients is promising.

### 2.8. ANRIL

As it is located in the genomic hot spot related to disease inheritance—namely, the CDKN2A/B locus—*ANRIL* has attracted widespread attention [83]. Past studies have shown that *ANRIL* was involved in the occurrence and development of a variety of cancers, including cancer susceptibility and the proliferation and migration process of cancer cells [84,85]. *ANRIL* rs1333048 has been confirmed to be a biomarker of breast cancer susceptibility [86]. In addition, rs4977574 and rs10757278 have also been proved to be associated with cancer risk. Studies have confirmed the importance of p16 as a regulator in cancer cells caused by cisplatin. With logistic regression analysis, Gong et al. found that *ANRIL* rs1333049 was associated with the low incidence of overall toxicity after severe cisplatin treatment in lung cancer patients (*p* = 0.028) [18]. According to previous studies, *ANRIL* regulates epigenetic silencing of *p14ARF* (or *p16INK4a*), which was an important regulator of cisplatin-induced apoptosis [87,88,89]. This may explain the functional changes associated with *ANRIL* polymorphisms that can affect the response to platinum-based chemotherapy in cancer patients. Compared with the paclitaxel-resistant group, Xu et al. found that the expression level of *ANRIL* was significantly increased in paclitaxel-sensitive lung adenocarcinoma cells. Similarly, *ANRIL* expression was higher after si-*ANRIL* transfection [90]. It shows that the expression level of *ANRIL* is related to paclitaxel resistance. Interestingly, rs10757278 SNP may be associated with *ANRIL* expression levels, as suggested by Huang et al. [86]. Based on the foregoing, it can be speculated that the SNP of rs10757278 affected cisplatin resistance. Further mechanistic studies revealed that *ANRIL* could significantly modulated the expression of apoptosis-related protein Bcl-2 protein and PARP protein [90]. In paclitaxel-sensitive lung adenocarcinoma cells, the expression of Bcl-2 was increased, but PARP was reduced. It indicates that *ANRIL* polymorphism-related abnormal expression may result in resistance to paclitaxel chemotherapy by regulating the expression of Bcl-2 and PARP. Another study explained the role of *ANRIL* in cisplatin resistance in ovarian cancer by down-regulating the expression of *let-7a* and then up-regulating the expression of *HMGA2* [91]. In lung cancer, the development of drug resistance is achieved by inhibiting the expression of miR-98 [92]. All these results indicate that *ANRIL* polymorphisms are related to the susceptibility to toxicity of cisplatin chemotherapy drugs, and its mechanism might be related to the polymorphisms affecting the centroid secondary structure and minimum free energy of *ANRIL* [39,93]. The role of *ANRIL* polymorphisms in chemotherapy response needs to be further investigated, especially in terms of mechanism. However, it cannot be denied that *ANRIL* polymorphisms have the potential to be biomarkers.

### 2.9. HOTAIR

*HOTAIR* is a lncRNA overexpressed in a variety of cancer cells, including lung cancer, hepatocellular carcinoma, and colorectal cancer. It is related to the prognosis of cancer patients [18,94]. A recent study extensively investigated the association of *HOTAIR* polymorphisms with different cancer risks [95]. The SNPs of rs4759314, rs920778, rs12826786, rs874945, and rs12427129 are all related to cancer risk. Through a study of 467 patients who received platinum chemotherapy, *HOTAIR* was overexpressed in cisplatin-resistant A549 cells, which were resistant to cisplatin by regulating p21 [26]. High expression of *HOTAIR* was observed in diffuse large B-cell lymphoma cells and correlated with chemoresistance [96]. It promoted cell growth and inhibited apoptosis by regulating H3K27me3 and activating the PI3K/AKT/NF-κB pathway, which was thought to be the mechanism by which *HOTAIR* regulates the resistance of large B-cell lymphoma to diffuse prednisone [97]. In addition, *HOTAIR* was thought to be involved in the resistance of diffuse large B-cell lymphoma to prednisone by regulating miR-130a [98]. Recent studies have shown that *HOTAIR* affects the chemotherapy resistance of patients with gastrointestinal stromal tumors by combining with miR-130a [99]. It is known that miR-130a-3p has the binding site of *ATG2B’s* 3′-UTR and encodes an autophagy-related protein [100], thereby regulating chemoresistance [101]. Moreover, *HOTAIR* rs7958904 may affect platinum-based chemotherapy by affecting the function or expression of *HOTAIR*. In addition, squamous cell carcinoma patients with *HOTAIR* rs7958904 were more likely to develop severe hematological toxicity after platinum-based chemotherapy. Even though the mechanism of its action needs further research to prove, we already know that *HOTAIR* polymorphisms were associated with cancer risk and the toxicity of cisplatin-based chemotherapy.

### 2.10. Other LncRNAs

In addition to the above, some other important lncRNAs have also been found to play an interesting role in chemotherapy resistance, and their expression levels are regulated by their polymorphisms. The expression of lncRNA *TP73 Antisense RNA 1* (*TP73-AS1*) is high in glioblastoma multiforme, and high expression is positively correlated with poor prognosis [102]. Further studies confirmed that the up-regulation of *TP73-AS1* promotes resistance of glioblastoma cancer stem cells to temozolomide by regulating the expression of metabolism-related genes and *aldehyde dehydrogenase 1 family member A1* (*ALDH1A1*). Rs3737589 G genotype carriers had higher expression levels of *TP73-AS1* than A genotype carriers [103]. Moreover, the *TP73-AS1* polymorphisms have also been revealed to be related to the risk of gastric cancer, in which rs3737589 SNP can be used as a potential biomarker for the prognosis of gastric cancer patients. Previous studies have shown that *Growth Arrest Specific 5* (*GAS5*) is a potential tumor suppressor [104] and is down-regulated in a variety of cancers [105,106,107]. Rs17359906 and rs1951625 G genotype carriers have a higher risk of recurrence than A genotype carriers after chemotherapy [108]. Subsequently, researchers found that the expression level of *GAS5* Rs55829688 with CC genotype carriers was significantly higher than that of TT and TC genotype carriers [109]. Recent studies have found that *GAS5*, as the endogenous “sponge” of miR-221-3p, participates in *ABCB1*-mediated adriamycin resistance of breast cancer by regulating the miR-221-3p/DKK2/WNT/β-catenin signaling pathway [110]. Similarly, lncRNA Nuclear Paraspeckle Assembly Transcript 1 (*NEAT1*) was indicated to be lowly expressed in nasopharyngeal carcinoma cells that are resistant to histone deacetylase inhibitors and could improve the resistance of nasopharyngeal carcinoma to histone deacetylase inhibitors by regulating the miR-129/Bcl-2 axis [111]. SNP of rs3825071 affects its expression level [112]. Although there is no direct conclusion to prove the direct effect of these lncRNAs polymorphisms on chemotherapy response, based on the existing evidence, we speculate that the above three lncRNAs polymorphisms also affect chemotherapy resistance and deserve further exploration.

## 3. Discussion

According to current research, lncRNAs SNPs can be used as potential biomarkers for cancer screening and diagnosis, and even as cancer treatment targets. Recent bioinformatics analysis has shown that *H19* can be used as an independent posterior factor of low-grade glioma [43]. In terms of cancer recurrence risk, lncRNA GAS5 rs17359906 and rs1951625 G genotype were significantly associated with high recurrence rates after chemotherapy [108]. The SNPs of *KCNQ1OT1* rs7128926 and rs7939976 have been shown to independently predict the recurrence-free survival and overall survival of gastric cancer patients [113]. Currently, chemotherapy is the main treatment method adopted by many cancer patients to prolong life and/or restore health. However, in many cancer patients receiving chemotherapy, there are still important unavoidable challenges, such as significant chemotherapy resistance, including intrinsic and acquired resistance [114]. Therefore, it is imperative to elucidate the mechanism of drug resistance and develop new immunosuppressants for cancer patients. Among chemotherapy resistance, most arise from acquired drug resistance, which is usually related to the expression of the *MDR1* gene [115]. The generation and degree of drug resistance vary among individuals, and small mutations in the expression of non-coding genes caused by lncRNAs polymorphisms may play an important role in this process. *H19* polymorphisms have been proven to regulate the methylation of the *MDR1* promoter in liver cancer cells, resulting in chemotherapy resistance [42]. In recent years, the direct or indirect regulation of the p53 tumor suppressor pathway by lncRNA has also attracted the attention of researchers. P53 participates in all steps of tumor initiation and development by regulating the expression of downstream genes, including DNA replication, transcription, and repair, thereby affecting the chemotherapy resistance of cancer cells [24]. Some lncRNAs polymorphisms also affect chemotherapy response by regulating PI3K/AKT/NF-κB, EMT and miRNA and by other ways (Figure 1).

Additionally, the toxicity of chemotherapy also seriously affects the patients’ survival and quality of life. Blood toxicity, gastrointestinal reactions, and liver and kidney function abnormalities all make cancer patients suffer psychological and physical stress in addition to cancer pain. Therefore, discovering more polymorphisms related to chemotherapy resistance and chemotherapy toxicity has an important practical significance for precise cancer treatment. Based on these study outcomes, lncRNAs are highly expressed in cancer cells, and their polymorphisms are the cause of their abnormal expression and/or structural abnormality. It then affects the function of its downstream genes or proteins, leading to changes in the sensitivity of cancer cells to drugs. It ultimately affects the response to chemotherapy, cancer progression, survival, and recurrence rates of cancer patients. In this review, our focus is limited to the impact of lncRNAs on cancer chemotherapy response and sensitivity since there are only a few studies on lncRNAs polymorphisms on cancer radiotherapy response or other physical therapy. In terms of the relevant mechanism, most of the content is not clear, and further exploration and confirmation are needed. There are also some lncRNAs that are also worthy of further exploration due to their close correlation between polymorphisms and expression levels, for example, *TP73-AS1*, *GAS5*, and *NEAT1*, even though we could not find direct evidence that their regulation of chemoresistance is affected by their polymorphisms.

## 4. Conclusions

In this review, we analyzed recently identified gene polymorphisms of lncRNAs affecting the response of chemotherapeutic drugs. Because the functional roles of these lncRNAs are largely unresolved, we have limited knowledge of the molecular mechanisms of their polymorphisms. The studies of *MIR2052HG*, *LINK-A*, *MEG3*, and *ANRIL* have revealed that their polymorphisms may regulate either the expression or the structure of functional lncRNAs, thereby exerting biological effects; however, more mechanisms are expected to be discovered based on detailed studies of RNA-protein and RNA–DNA interactions. In conclusion, the polymorphisms of lncRNAs may serve as a biomarker for predicting the response of cancer patients to chemotherapy. Clinically, suitable chemotherapy drugs can be selected according to the polymorphisms of different patients.

## Figures and Tables

**Table 1:** Known lncRNA polymorphisms affecting drug response in cancer therapy.

LncRNA	Polymorphisms	Cancer Type	Patient Population	Drug	Effect	Reference
*MIR2052 Host Gene* (*MIR2052HG*)	rs4476990 and rs3802201	Breast cancer	4658 women with breast cancer, including 252 women experiencing a breast cancer recurrence	Aromatase Inhibitor (AIs)	Regulated ERα expression in the presence of AIs	[21]
			4406 controls without recurrence of breast cancer and 252 cases with recurrence			[22]
*Maternally Expressed 3*(*MEG3*)	rs10132552	Breast cancer	144 women with locally advanced invasive breast cancer	Paclitaxel and cisplatin	Associated with good DFS and PCR rate	[23]
		Nasopharyngeal carcinoma	505 newly diagnosed nasopharyngeal carcinoma patients	Platinum-based chemotherapy drug	Associated with treatment response and risk of developing anemia	[24]
	rs941576	Breast cancer	144 women with locally advanced invasive breast cancer	Paclitaxel and cisplatin	Associated with good DFS	[23]
	rs116907618	Lung cancer	467 lung cancer patients	Platinum-based chemotherapy drug	Associated with severe gastrointestinal toxicity	[18]
*H19 Imprinted Maternally Expressed Transcript*(*H19*)	rs2839698, rs3842761, rs4244809, rs7924316, rs4244809	Epithelial ovarian cancer (EOC)	43 platinum-resistant and 138 platinum-sensitive EOC patients	Platinum-based chemotherapy drug	Associated with platinum-based chemoresistance	[25]
	rs2104725	Lung cancer	467 lung cancer patients	Platinum-based chemotherapy drug	Associated with severe gastrointestinal toxicity	[18]
	rs2839698				Associated with severe gastrointestinal or hematologic toxicities	
*antisense non-coding RNA in the INK4 locus* (*ANRIL*)	rs1333049	Lung cancer	467 lung cancer patients	Platinum-based chemotherapy drug	Associated with the incidence of severe gastrointestinal toxicity	[18]
	rs10120688				Associated with severe hematologic toxicity	
*HOX Transcript Antisense RNA* (*HOTAIR*)	rs7958904	Lung cancer	467 lung cancer patients	Platinum-based chemotherapy drug	Associated with the incidence of severe gastrointestinal toxicity	[18]
	rs1899663				Associated with severe gastrointestinal toxicity in age ≥ 57	
*metastasis-associated with lung adenocarcinoma transcript-1* (*MALAT1*)	rs619586	Lung cancer	467 lung cancer patients	Platinum-based chemotherapy drug	Associated with gastrointestinal toxicity	[18]
	rs3200401	Metastatic colorectal cancer	98 colorectal cancer patients	Irinotecan	Associated with Pb derived toxicity and tumor resistance to irinotecan	[26]
*cancer susceptibility candidate 8* (*CASC8*)	rs10505477	Lung Cancer	498 lung cancer patients and healthy controls	Platinum-based chemotherapy drug	Associated with platinum-based chemotherapy response and toxicity	[27]
*Long Intergenic Non-Protein Coding RNA 1139*(*LINK-A*)	rs12095274	Breast cancer	Breast cancer patients	AKT inhibitors	Leading to resistance to AKT inhibitors	[19]
*Long intergenic non-protein coding RNA-regulator of reprogramming* (*Linc-ROR*)	rs2027701	Nasopharyngeal carcinoma	505 newly diagnosed nasopharyngeal carcinoma patients	Platinum-based chemotherapy drug	Associated with chemoresistance and toxicity	[24]

## Data Availability

Not applicable.
